# Proteomic Analysis Reveals the Positive Effect of Exogenous Spermidine in Tomato Seedlings' Response to High-Temperature Stress

**DOI:** 10.3389/fpls.2017.00120

**Published:** 2017-02-06

**Authors:** Qinqin Sang, Xi Shan, Yahong An, Sheng Shu, Jin Sun, Shirong Guo

**Affiliations:** ^1^Key Laboratory of Southern Vegetable Crop Genetic Improvement in Ministry of Agriculture, College of Horticulture, Nanjing Agricultural UniversityNanjing, China; ^2^Nanjing Agricultural University (Suqian), Academy of Protected HorticultureSuqian, China

**Keywords:** antioxidant, high-temperature stress, proteomics, spermidine, tomato

## Abstract

Polyamines are phytohormones that regulate plant growth and development as well as the response to environmental stresses. To evaluate their functions in high-temperature stress responses, the effects of exogenous spermidine (Spd) were determined in tomato leaves using two-dimensional electrophoresis and MALDI-TOF/TOF MS. A total of 67 differentially expressed proteins were identified in response to high-temperature stress and/or exogenous Spd, which were grouped into different categories according to biological processes. The four largest categories included proteins involved in photosynthesis (27%), cell rescue, and defense (24%), protein synthesis, folding and degradation (22%), and energy and metabolism (13%). Exogenous Spd up-regulated most identified proteins involved in photosynthesis, implying an enhancement in photosynthetic capacity. Meanwhile, physiological analysis showed that Spd could improve net photosynthetic rate and the biomass accumulation. Moreover, an increased high-temperature stress tolerance by exogenous Spd would contribute to the higher expressions of proteins involved in cell rescue and defense, and Spd regulated the antioxidant enzymes activities and related genes expression in tomato seedlings exposed to high temperature. Taken together, these findings provide a better understanding of the Spd-induced high-temperature resistance by proteomic approaches, providing valuable insight into improving the high-temperature stress tolerance in the global warming epoch.

## Introduction

High-temperature stress represents one of the most frequent abiotic stresses worldwide, inducing several physiological and biochemical processes in cells, and limiting the growth and productivity of plants (Bita and Gerats, 2013). Plants respond to high temperature by reprogramming their proteome, metabolome and transcriptome to establish a new steady-state balance of metabolic processes (Kosová et al., 2011; Lin H. H. et al., 2015; Sruthi et al., 2016).

Polyamines (PAs) are ubiquitous low-molecular-weight aliphatic amines, and include putrescine (Put), spermidine (Spd), and spermine (Spm). PAs are known to participate in the regulation of physiological and developmental processes (Liu et al., 2007; Gupta et al., 2013), and they are also involved in the defense reaction of plants against various environmental stresses (Todorova et al., 2007; Berberich et al., 2015; Pál et al., 2015). The integration of environmental stimuli, signal transduction and the stress response is mediated, at least partially, by intensive cross-talk among plant hormones (Wahid et al., 2007). Recent studies indicated that polyamines act as cellular signals in the intricate cross talk with different metabolic routes and complex hormonal pathways (Pál et al., 2015). The exogenous Spd enhancement of high-temperature stress tolerance via the involvement of antioxidant ability and photosynthetic efficiency had been described (Tian et al., 2012; Mostofa et al., 2014), but little information about Spd regulating proteomic changes under the high-temperature stress is available.

As mRNA abundance is not enough to provide information about the proteins, proteomic analysis has become a powerful tool to elucidate the mechanisms of plant stress tolerance (Skalák et al., 2016). Previous studies reported that PAs could bind to charged spots at protein interfaces and modulate electrostatic protein–protein interactions to regulate the protein functions (Berwanger et al., 2010). Exogenous polyamines had been found to activate multiple pathways that conferred increased salt and drought tolerances in bermudagrass by reprogramming the proteome (Shi et al., 2013). Li et al. (2013) and Yuan et al. (2016) showed that application of Spd/Put changed the expression of proteins and contributed to counteract the damage induced by salt stress in cucumber seedlings. Igarashi and Kashiwagi (2015) reported that polyamines could stimulate the synthesis of proteins at the translation level due to the formation of a polyamine-RNA complex.

The tomato *(Lycopersicon esculentum*) is one of the most important vegetables from both the nutritional and economic points of view. The effects of exogenous Spd in enhancing the stress tolerance had been described in cucumber (Tian et al., 2012) and in rice (Mostofa et al., 2014). However, little information is available to explain the specific mechanisms by which PAs regulate the high-temperature stress responses through a proteomic approach. In this study, we investigated the differentially expressed proteins in tomato leaves through 2-dimensional gel electrophoresis to better understand the underlying mechanisms of Spd application in high-temperature stress resistance.

## Materials and methods

### Plant materials and treatments

Tomato (*Lycopersicon esculentum* Mill. cv. Puhong 968) seeds were obtained from the Shanghai Academy of Agricultural Sciences, China. Seeds were germinated and grown in plastic nutrition pots filled with growth media (Zhenjiang Peilei Co., Ltd., China). The germinated seedlings were grown under controlled condition (light intensity, 600 μmol m^−2^·s^−1^; day/night temperature, 25/18°C; light/dark photoperiod, 14 h/10 h; relative humidity, 55–65%) in growth chambers (Ningbo Jiangnan Instrument Factory, Ningbo, China).

After the third true leaf developed, the seedlings were subjected to high-temperature (day/night temperature, 38/28°C; light/dark photoperiod, 14/10 h; relative humidity, 55–65%). The experimental plots included four different treatments: (1) Cont; (2) Spd (1 mM); (3) HT; (4) HT+ Spd (1 mM). The concentration of Spd was selected on the basis of previous experiment (data not shown). One millimole Spd was sprayed to leaves at 17:00 every day, and the control plants were sprayed with distilled water. After 7 days of treatment, the third fully expanded tomato leaves of each treatment were stored at −80°C for physiological and proteomic analysis. The experiment was arranged in a randomized complete block design and biological replicates were independently carried out three times.

### Measurement of dry weight, chlorophyll content, and net photosynthetic rate (Pn)

The tomato seedlings were washed with sterile distilled water. After wiped with gauze, samples were dried in an oven at 105°C for 15 min followed by 75°C for 72 h, until reaching a constant weight, and then weighed for dry weight. Chlorophyll was extracted with a mixture of acetone, ethanol and water (4.5: 4.5: 1 by volume) and its content was estimated using the method of Arnon (1949). Pn was measured using a portable photosynthesis system (LI-6400, LI-COR Inc, USA).

### Protein extraction

Protein extraction was performed according to a modified version of the trichloroacetic acid (TCA) acetone precipitation method described by Hurkman and Tanaka (1986). Frozen leaf tissues were ground in liquid nitrogen and suspended in ice-cold extraction buffer (8 M urea, 1% (w/v) dithiothreitol (DTT), 4% (w/v) CHAPS and 40 mM Tris). Then the homogenates were centrifuged at 15,000 × g for 20 min at 4°C, and the supernatants were precipitated overnight with ice-cold acetone containing 10% (w/v) TCA and 0.07% (v/v) β-mercaptoethanol. The resulting protein-containing suspensions were centrifuged at 20,000 × g for 30 min at 4°C, and then the protein pellets were washed three times with cold acetone containing 0.07% (v/v) β-mercaptoethanol. Finally, the protein pellets were air-dried at room temperature and dissolved in rehydration buffer (8 M urea, 1 M thiourea, 2% w/v CHAPS). The protein concentrations were determined by the methods of Bradford (1976) using bovine serum albumin as the standard, and then the protein was stored at −80°C until being subjected to two-dimensional gel electrophoresis (2-DE).

### 2-DE

For first dimensional isoelectric focusing (IEF), IPG strips (GE Healthcare, San Francisco, CA, USA, 17 cm, pH 4–7 linear gradient) were used according to the methods of Li et al. (2013). The dry IPG strips were rehydrated at room temperature for 12–16 h in 350 μL rehydration solution [8 M (w/v) urea, 1 M (w/v) thiourea, 2% (w/v) CHAPS, 65 mM DTT, 0.8% (v/v) IPG buffer 4–7, and 1% (w/v) bromophenol blue)]. Following rehydration, the IPG strips were run on an Ettan IPGphor 3 (GE Healthcare, USA) with a gradient of 100 V (1 h), 200 V (1 h), 200 V (1 h), 500 V (1 h), 1000 V (1 h), 4000 V (1 h), and 10,000 V (1 h), finally reaching a value of 75,000 V h. The working temperature was maintained at 20°C with a maximum current of 50 mA per strip. After the first dimension, the IEF strips were equilibrated for 15 min in equilibration solution I [1% (w/v) DTT, 6 M urea, 30% (v/v) glycerol, 2% (w/v) SDS, and 50 mM Tris–HCl (pH 8.8)], and then in equilibration solution II [2.5% (w/v) iodoacetamide, 6 M urea, 30% (v/v) glycerol, 2% (w/v) SDS, and 50 mM Tris–HCl (pH 8.8)] for 15 min.

The second dimensional SDS-polyacrylamide gel electrophoresis was performed on running gels (Hoefer SE600 Ruby Standard Vertical System, GE Healthcare; 12.5% polyacrylamide) as described by Laemmli (1970). The strips were embedded on the top of the SDS gel and then sealed with 1% molten agarose solution. Electrophoresis was carried out at 15 mA per gel until the bromophenol blue dye reached the bottom of the gel. After the 2-DE, the gels were stained overnight with Coomassie Brilliant Blue (CBB) R-250 solution (0.1% (w/v) of CBB R-250 in 1:4:5 (v/v) methanol: acetic acid: deionized water) and destained with a 1:1:8 (v/v) methanol: acetic acid: deionized water solution with several changes, until a colorless background was achieved.

### Image and data analysis

The 2-D gels were scanned with an Image Scanner III (GE Healthcare, San Francisco, USA). Spot detection, quantification and matching were performed with Imagemaster™ 2D Platinum software (version 6.0, GE Healthcare, San Francisco, USA). The intensity of each spot on the 2-D gels was quantified based on the volumes percentage (vol. %). Only spots with significant changes (at least 1.5-fold quantitative changes, *P* < 0.05) were considered to be differentially expressed.

### Protein identification

The protein spots were excised from the polyacrylamide gels, and identified using MALDI-TOF/TOF MS by an Ultraflex II mass spectrometer (Applied Biosystems, Foster City, CA, USA). The resulting peptide mass lists were searched in NCBI (http://www.ncbi.nlm.nih.gov) using the software MASCOT version 2.1 (Matrix Science, London, UK). The parameter criteria were as follows: trypsin cleavage, one missed cleavage allowed; carbamidomethyl (C) set as a fixed modification; oxidation of methionines allowed as a variable modification; peptide mass tolerance within 100 ppm; fragment tolerance set to ± 0.4 Da; and minimum ion score confidence interval for MS/MS data set to 95%.

The classification of the identified proteins was performed by searching in the UniProt Knowledgebase (UniProtKB, http://www.uniprot.org).

### Hierarchical cluster analysis and interaction network

The hierarchical clustering of the protein expression patterns was performed on the log_2_ transformed vol. % of each protein spot using Cluster software (version 3.0). The complete linkage algorithm was enabled, and the results were plotted using Treeview software (version 1.60).

Mapping of the interaction network was performed using the STRING database (http://string.embl.de) based on conformed and predicted interactions.

### Enzyme activity analysis

Ascorbate peroxidase (APX, EC 1.11.1.11) activity was determined according to Nakano and Asada (1981) by measuring the rate of ascorbate oxidation at 290 nm (ε = 2.8 mM^−1^ cm^−1^). Dehydroascorbate reductase (DHAR, EC 1.8.5.1) activity was calculated from the change in absorbance at 265 nm and the extinction coefficient of 14 mM^−1^ cm^−1^, as described by Nakano and Asada (1981). Superoxide dismutase (SOD, EC 1.15.1.1) activity was calculated by inhibiting the photochemical reduction of NBT at 560 nm. One unit of SOD activity was defined as the amount of enzyme that caused 50% inhibition of NBT reduction rate (Becana et al., 1986).

### Total RNA extraction and quantitative real-time PCR (qRT-PCR) analysis

The total RNA was extracted from the tomato leaf tissues as described in the TRI reagent protocol (Takara Bio Inc.). The total RNA and cDNA syntheses were performed according to the manufacturer's instructions. The primers were designed according to the NCBI (Supplementary Table 1). qRT-PCR was performed with the SYBR PrimeScript™ RT-PCR Kit (Takara Bio Inc.) according to the manufacturer's instructions. All experiments were repeated three times and the relative gene expression was calculated by the 2^−ΔΔCt^ method.

### Statistical analysis

All biochemical analyses were conducted at least three times. Data were statistically analyzed with statistical software SPSS 17.0 (SPSS Inc., Chicago, IL, USA) using Duncan's multiple range test at the *P* < 0.05 level of significance.

## Results

### Morphological and physiological responses

**Figure 1:**
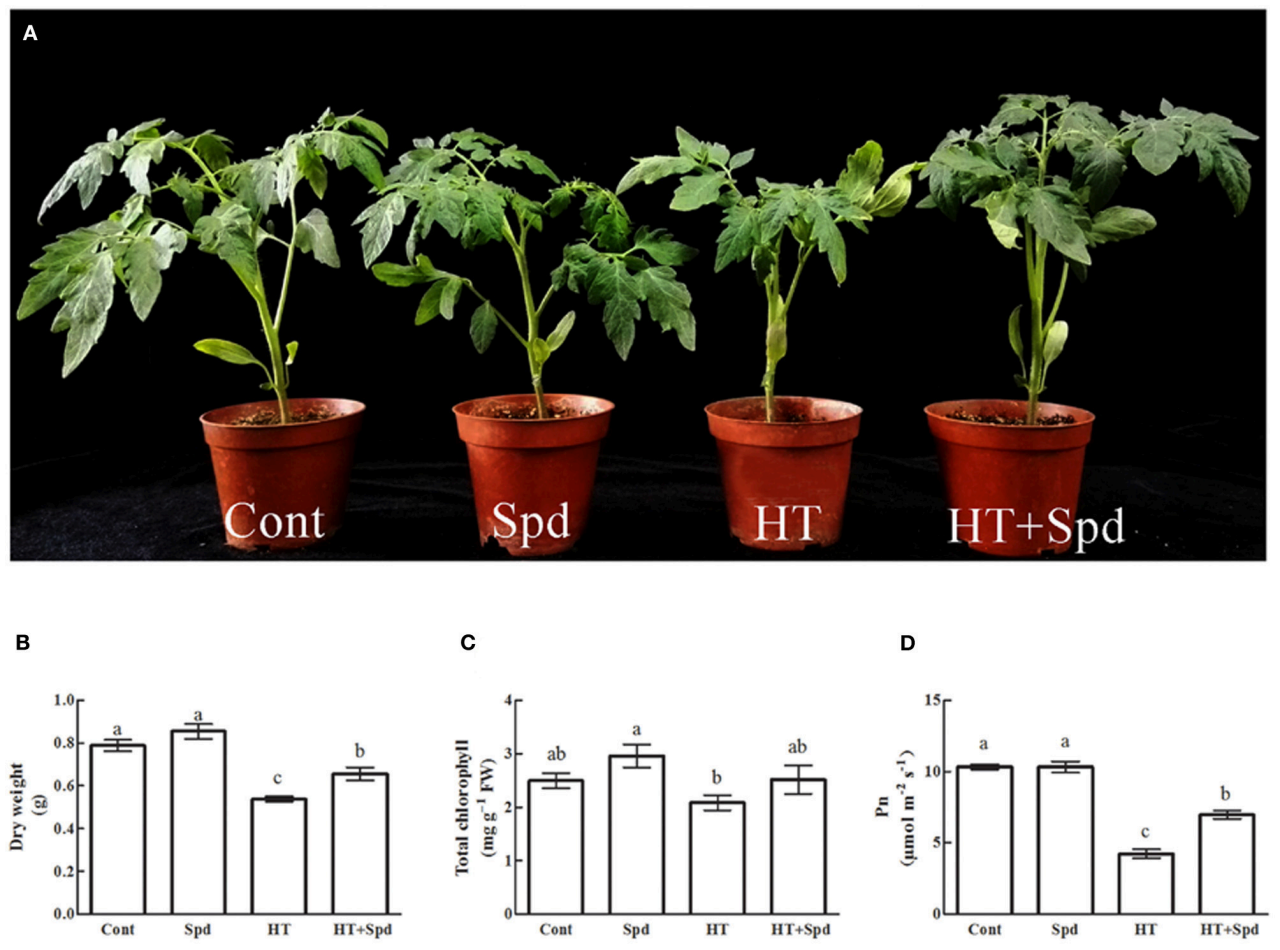
**Effects of Spd on plant morphology (A)**, dry weight **(B)**, chlorophyll content **(C)**, and Pn **(D)** in leaves of tomato exposed to high temperature stress. Cont, control plants under 25/18°C (day/night); Spd, plants under 25/18°C with 1 mM Spd foliar spraying; HT, plants under 38/28°C; HT+Spd, plants under 38/28°C with 1 mM Spd foliar spraying. Each histogram represents a mean ± SE of three independent experiments (*n* = 3). Different letters indicate significant differences between treatments (*P* < 0.05) according to Duncan's multiple range tests.

After 7 days' treatment with exogenous Spd, no significant differences were observed in the tomato leaves under non-stressful conditions. Phenotypic observations showed that the untreated high-temperature stressed seedlings exhibited chlorosis and yellowing, whereas the Spd-treated seedlings had a better visual appearance (Figure 1A). Under the high-temperature stress, the dry weight, chlorophyll content and net photosynthetic rate (Pn) decreased by 33.0, 16.4, and 58.9%, respectively. However, exogenous Spd application resulted in improvements in these parameters (Figure 1).

### Proteomic analysis

**Figure 2:**
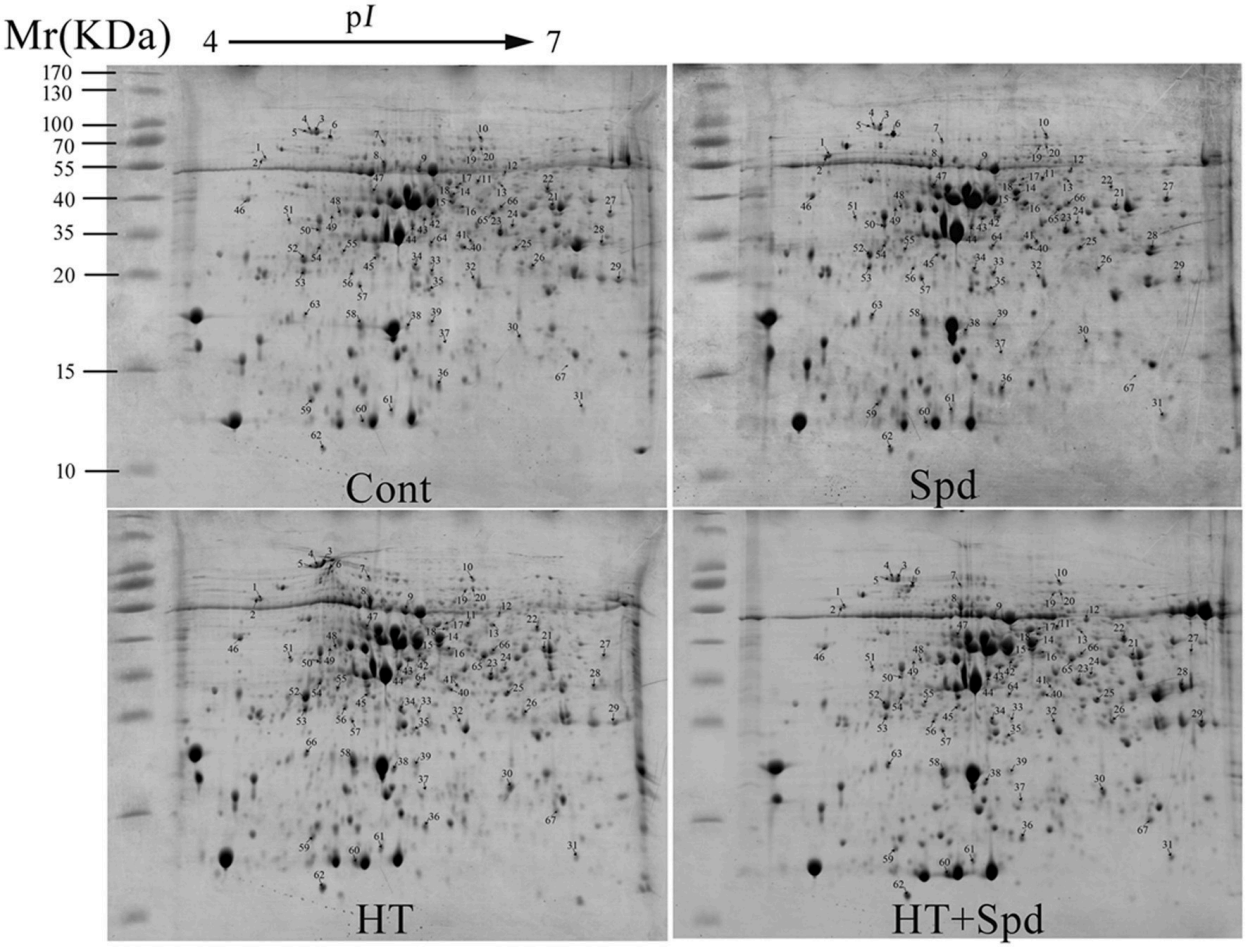
**Coomassie Brilliant blue (R-250)-stained 2-DE gels**. Spot numbers indicate the 67 identified differentially expressed proteins. The range of the molecular mass of protein markers was from 10 to 170 kDa.

**Figure 3:**
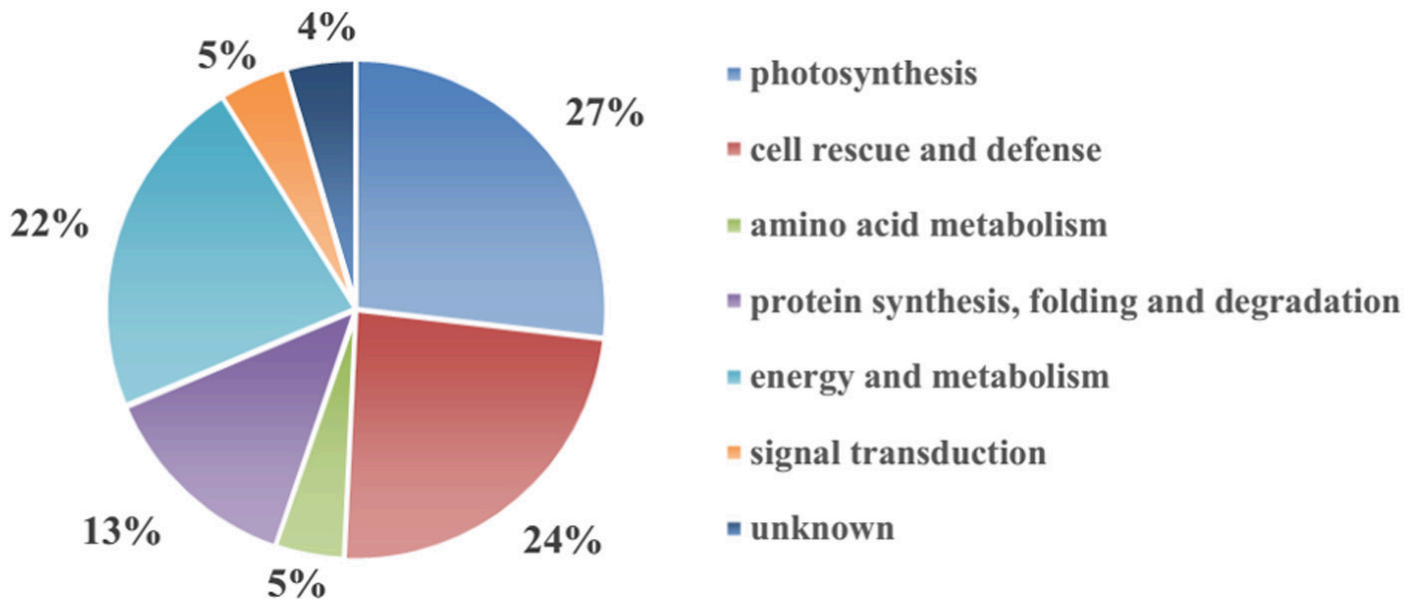
**Functional classification of the 67 identified differentially expressed proteins in tomato leaves**.

To reveal the protective effect of exogenous Spd on the tomato under high-temperature stress, a total of 67 differentially expressed spots were identified using 2-DE and MALDI-TOF-MS (Figure 2, Table 1, Supplementary Figure 1). To better understand which physiological process was regulated by Spd under the high-temperature stress, the identified proteins were grouped into 7 categories based on their biological functions according to Gene Ontology (Figure 3). Among the 67 proteins, the majority were sorted into photosynthesis (27%), followed by cell rescue and defense (24%), protein synthesis, folding, and degradation (22%), energy and metabolism (13%), amino acid metabolism (5%), signal transduction (5%), and unknown (4%).

**Table 1:** **Leaf proteins responsive to high temperature stress and/or Spd identified by MALDI-TOF/TOF MS**.

**^a^Spot No**.	**Protein name**	**Accession No**.	**^b^TpI/EpI**	**^c^TMr/EMr (kDa)**	**Score**	**^d^MP**	**^e^Cov (%)**	**Foldchanges**			
								**Cont/Cont**	**Spd/Cont**	**HT/Cont**	**HT+Spd/HT**
**PHOTOSYNTHESIS**											
6	ruBisCO large subunit-binding protein subunit alpha	gi|460411525	5.21/4.90	62.03/69	313	17	37.24	1.00	2.02	0.28	2.92
11	glutamate 1-semialdehyde 2,1-aminomutase	gi|642911	6.54/5.84	51.72/46	858	18	56.13	1.00	0.90	1.51	0.62
13	glutamate 1-semialdehyde 2,1-aminomutase	gi|642911	6.54/5.99	51.72/45	361	15	47.61	1.00	1.12	1.68	0.65
14	ribulose bisphosphate carboxylase/oxygenase activase 1, chloroplastic isoform X1	gi|460401823	8.15/5.68	49.05/40	1070	26	70.52	1.00	1.54	2.96	0.88
15	ribulose bisphosphate carboxylase/oxygenase activase 1, chloroplastic isoform X1	gi|460401823	8.15/5.69	49.05/39	1120	28	73.92	1.00	1.07	1.89	0.74
16	Ribulose bisphosphate carboxylase/oxygenase activase, chloroplastic-like	gi|723739979	8.76/5.74	50.97/38	481	16	38.56	1.00	1.06	0.65	1.31
23	ferredoxin–NADP reductase, leaf-type isozyme, chloroplastic	gi|460373374	8.37/5.99	40.77/34	617	18	55.25	1.00	0.90	0.67	1.23
26	carbonic anhydrase	gi|56562177	6.67/6.21	34.84/25	563	16	63.55	1.00	0.72	0.80	1.29
28	ribulose 1,5-bisphosphate carboxylase/oxygenase large subunit (chloroplast)	gi|779776586	6.55/6.67	53.43/33	623	24	54.30	1.00	0.81	0.60	1.65
32	oxygen-evolving enhancer protein 1, chloroplastic	gi|823630968	5.91/5.83	35.15/25	582	13	52.89	1.00	1.20	0.00	+
39	oxygen-evolving enhancer protein 2, chloroplastic	gi|929045135	7.63/5.54	27.86/19	352	8	42.25	1.00	0.85	0.47	1.85
43	coproporphyrinogen-III oxidase 1, chloroplastic	gi|460405900	5.92/5.48	45.24/37	744	21	58.40	1.00	1.21	1.59	0.87
49	ribulose 1,5-bisphosphate carboxylase, partial (chloroplast)	gi|488453392	6.99/4.90	48.29/37	189	8	28.24	1.00	1.05	0.55	1.94
57	oxygen-evolving enhancer protein 1, chloroplastic	gi|823630968	5.91/5.09	35.15/24	692	14	56.53	1.00	1.32	0.52	1.61
60	ribulose-1,5-bisphophate carboxylase/ oxygenase small subunit	gi|170500	3.67/5.13	20.45/12	319	9	55.00	1.00	0.59	1.36	1.31
61	photosystem II reaction center Psb28 protein	gi|460403300	9.42/5.30	20.25/13	121	5	31.67	1.00	1.67	2.51	0.61
62	ribulose-1,5-bisphosphate carboxylase/oxygenase large subunit, partial (chloroplast)	gi|778481335	6.18/4.86	5.68/11	84	3	58.82	1.00	1.42	2.69	0.83
64	ribulose bisphosphate carboxylase/oxygenase activase, chloroplastic-like	gi|723739979	8.76/5.53	50.97/31	771	21	39.65	1.00	0.69	0.48	1.23
**CELL RESCUE AND DEFENSE**											
3	heat shock protein 70	gi|158635118	5.41/4.80	74.41/78	388	22	33.67	1.00	0.86	1.43	0.60
4	stromal 70 kDa heat shock-related protein, chloroplastic	gi|460369188	5.20/4.77	74.96/75	1230	33	54.48	1.00	0.76	1.86	0.49
5	stromal 70 kDa heat shock-related protein, chloroplastic	gi|460369188	5.20/4.74	74.96/77	1090	27	47.08	1.00	0.99	1.90	0.41
25	stromal ascorbate peroxidase	gi|807201017	8.48/6.11	38.07/29	945	23	76.81	1.00	1.13	1.56	0.94
29	dehydroascorbate reductase	gi|929524249	6.32/6.79	23.71/24	578	17	83.33	1.00	1.02	0.58	1.86
30	temperature-induced lipocalin'	gi|77744859	5.96/6.15	21.30/17	436	10	60.00	1.00	0.87	3.16	0.78
35	superoxide dismutase [Fe] (plastid)	gi|33413303	6.60/5.52	27.89/23	131	5	20.08	1.00	0.93	0.50	1.54
36	superoxide dismutase [Cu-Zn], chloroplastic	gi|915409259	6.02/5.62	22.38/14	760	6	58.53	1.00	1.08	0.64	1.06
37	class I small heat shock protein	gi|349591296	5.57/5.59	17.62/16	551	12	73.38	0.00	0.00	+	1.74
41	thioredoxin-like protein CDSP32, chloroplastic	gi|460385401	7.57/5.81	33.78/32	175	12	39.19	1.00	1.15	0.49	1.73
44	2-oxoglutarate-dependent dioxygenase homolog, partial	gi|717140	6.82/5.40	25.86/36	518	11	43.61	1.00	0.65	1.36	0.79
45	plasma membrane-associated cation-binding protein 1	gi|460405902	5.03/5.20	21.98/28	275	12	73.63	1.00	1.22	2.55	0.57
58	23 kda heat-induced protein {N-terminal}	gi|1835994	3.75/5.10	27.86/19	134	1	87.50	1.00	1.19	1.77	0.67
59	inducible plastid-lipid associated protein	gi|75266304	5.81/4.79	18.30/13	391	8	70.69	1.00	0.98	1.41	0.65
63	2-Cys peroxiredoxin BAS1, chloroplastic	gi|460407951	6.00/4.74	29.73/20	87	3	10.11	1.00	1.92	1.96	0.48
67	class II small heat shock protein Le-HSP17.6	gi|1773291	6.32/6.46	17.67/15	191	7	53.80	0.00	0.00	+	1.41
**AMINO ACID METABOLISM**											
47	glutamine synthetase, chloroplastic	gi|460367196	6.29/5.16	47.85/41	552	17	40.74	1.00	1.15	0.48	1.42
48	cysteine synthase, chloroplastic/chromoplastic	gi|460398434	5.41/4.96	41.26/37	900	12	46.89	1.00	0.38	0.26	1.94
50	serine carboxypeptidase-like 20	gi|460393680	5.43/4.83	56.46/36	211	4	11.04	1.00	1.08	0.55	1.63
**PROTEIN SYNTHESIS, FOLDING AND DEGRADATION**											
7	ATP-dependent zinc metalloprotease FTSH 2, chloroplastic	gi|460395390	6.00/5.22	74.42/69	770	25	51.37	1.00	0.91	0.60	1.44
17	elongation factor TuB, chloroplastic-like	gi|460391817	6.69/5.72	56.29/46	98	10	22.97	1.00	0.74	0.58	1.18
20	putative inosine monophosphate cyclohydrolase	gi|260528216	6.21/5.87	66.20/66	269	16	31.67	1.00	0.81	0.69	1.49
33	proteasome subunit alpha type-2-A-like	gi|460405457	5.39/5.54	25.66/26	524	12	71.06	1.00	1.07	0.34	1.72
38	peptidyl-prolyl cis-trans isomerase FKBP16-3, chloroplastic	gi|460381848	6.75/5.37	25.76/18	324	8	30.64	1.00	1.25	1.59	0.80
46	ankyrin repeat domain-containing protein 2	gi|460369292	4.43/4.33	37.35/39	745	16	56.73	1.00	1.02	1.43	0.82
52	cysteine proteinase 3-like	gi|460396286	5.33/4.73	39.63/28	297	9	43.18	1.00	0.89	1.89	0.63
54	haloacid dehalogenase-like hydrolase domain-containing protein At3g48420	gi|460381143	5.67/4.83	34.50/31	697	17	58.04	1.00	0.92	1.34	0.73
66	mRNA binding protein precursor	gi|936975812	7.1/6.00	44.06/38	650	16	47.42	1.00	1.21	0.80	1.41
**ENERGY AND METABOLISM**											
8	ATP synthase CF1 alpha subunit (chloroplast)	gi|779776563	5.14/5.22	55.43/56	843	20	45.96	1.00	0.93	1.45	0.63
9	ATP synthase CF1 beta subunit (chloroplast)	gi|779776585	5.28/5.43	53.49/51	1560	26	75.30	1.00	1.08	1.93	0.66
10	transketolase, chloroplastic	gi|460406209	5.97/5.87	80.27/70	421	23	39.35	1.00	0.77	0.55	1.53
18	phosphoglycerate kinase, chloroplastic	gi|460396820	7.66/5.73	50.59/43	808	27	74.90	1.00	0.94	1.00	1.56
19	2,3-bisphosphoglycerate-independent phosphoglycerate mutase	gi|460396104	5.59/5.83	61.28/65	535	29	64.94	1.00	0.83	0.53	1.60
21	mitochondrial malate dehydrogenase	gi|927442679	8.73/6.34	36.29/38	642	12	50.58	1.00	0.94	0.68	1.28
22	glyceraldehyde-3-phosphate dehydrogenase B, chloroplastic-like	gi|460415552	6.72/6.28	48.54/41	459	16	37.33	1.00	0.84	0.49	2.05
24	fructose-bisphosphate aldolase 1, chloroplastic	gi|808175957	8.15/6.09	42.66/36	684	15	51.79	1.00	0.80	1.58	0.52
27	fructose-bisphosphate aldolase, cytoplasmic isozyme 1	gi|840084522	6.86/6.73	38.41/38	755	13	52.66	1.00	1.05	0.53	1.96
31	nucleoside diphosphate kinase	gi|575953	6.84/6.60	15.47/13	608	8	46.48	1.00	1.15	1.79	1.05
34	triosephosphate isomerase, chloroplastic	gi|460370086	6.45/5.45	35.04/25	769	19	70.55	1.00	1.09	1.71	0.88
42	fructose-bisphosphate aldolase 1, chloroplastic-like	gi|460375513	6.07/5.55	42.87/37	816	15	47.59	1.00	0.97	0.48	1.22
53	ATP synthase beta subunit, partial (chloroplast)	gi|159227612	5.18/4.73	35.93/26	102	8	35.71	1.00	0.76	1.31	0.50
55	ribose-5-phosphate isomerase 3, chloroplastic	gi|460368501	6.00/4.95	31.19/31	458	6	28.33	1.00	1.11	0.65	0.99
65	malate dehydrogenase	gi|460404529	5.91/5.94	35.70/38	128	8	25.00	1.00	0.80	1.51	0.95
**SIGNAL TRANSDUCTION**											
1	calreticulin	gi|460368893	4.50/4.45	47.80/56	412	21	42.69	1.00	1.46	2.66	0.64
2	calreticulin	gi|460368893	4.50/4.42	47.80/56	439	20	52.28	1.00	2.38	1.97	0.53
56	harpin binding protein 1	gi|38679319	6.25/5.04	30.29/25	643	13	55.43	1.00	0.81	0.66	0.99
**UNKNOWN**											
12	Hop-interacting protein THI113	gi|365222922	5.82/6.04	37.34/50	507	13	69.14	1.00	1.26	0.82	2.47
40	unnamed protein product	gi|939066554	5.64/5.76	21.84/31	151	10	46.94	1.00	1.09	0.73	1.32
51	uncharacterized protein LOC101260160	gi|460398472	4.66/4.64	35.10/36	164	15	52.85	1.00	0.92	1.27	0.60

a *Spot numbers corresponding to spots in Figure 1*.

b *TpI and EpI are the theoretical isoelectric point and experimental isoelectric point, respectively*.

c *TMr and EMr are the theoretical molecular mass and experimental molecular mass, respectively*.

d *The total number of identified peptides*.

e *Percentage of sequence coverage by matched peptides*.

**Figure 4:**
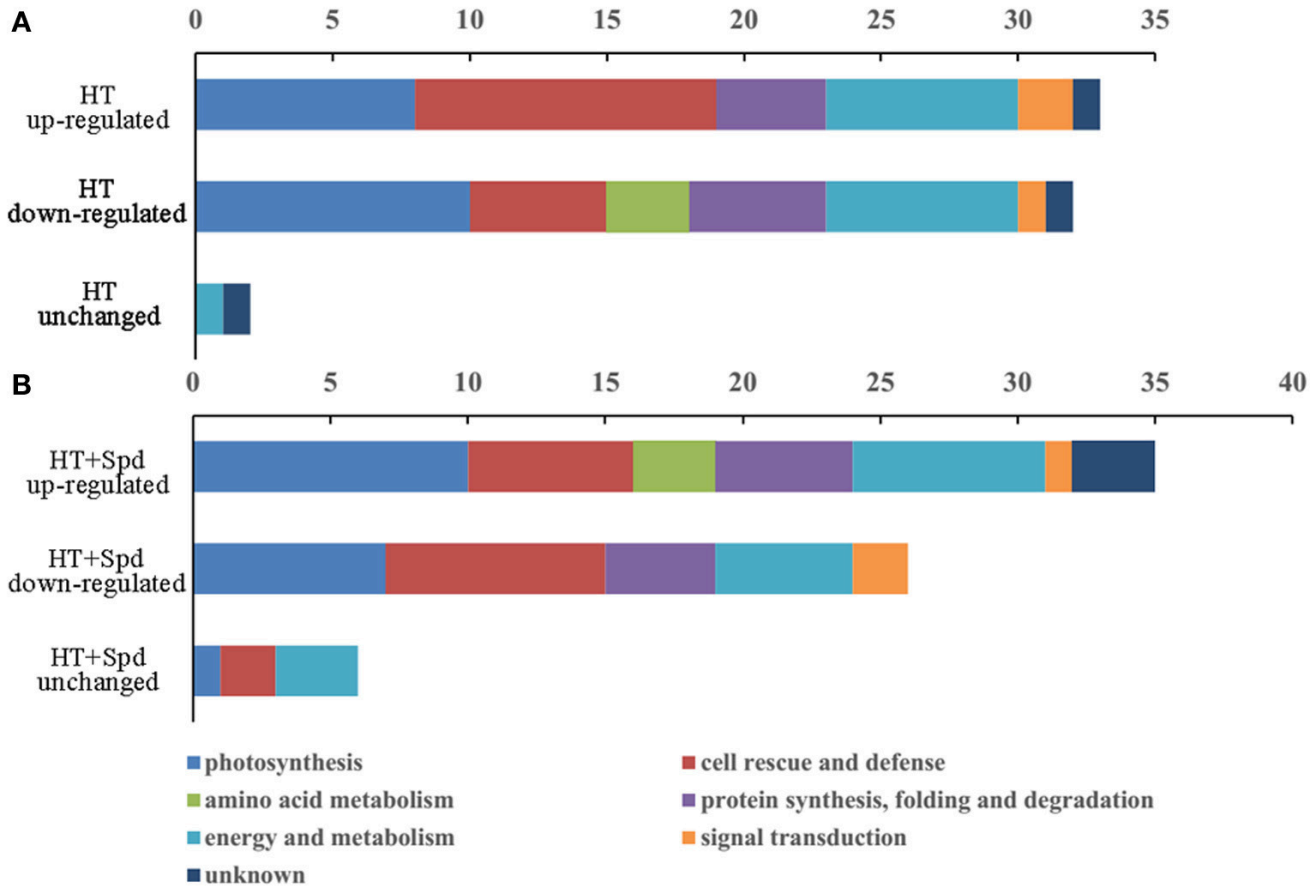
**The number and functional classification of identified proteins changed in abundance in tomato leaves. (A)** Differentially expression proteins responded to high temperature (HT) stress compare with the control. **(B)** Differentially expression proteins responded to Spd under high temperature stress (HT+Spd) compare with high temperature stress alone.

Compared with the control, there were 33 up-regulated spots and 32 down-regulated spots in response to the high-temperature stress (Figure 4A). For the high-temperature stress induced proteins, the most highly enriched category was cell rescue and defense. However, exogenous Spd up-regulated 35 spots and down-regulated 26 spots compared with the untreated seedlings subjected to high-temperature stress, and of these proteins, the most prevalent category was photosynthesis (Figure 4B).

**Figure 5:**
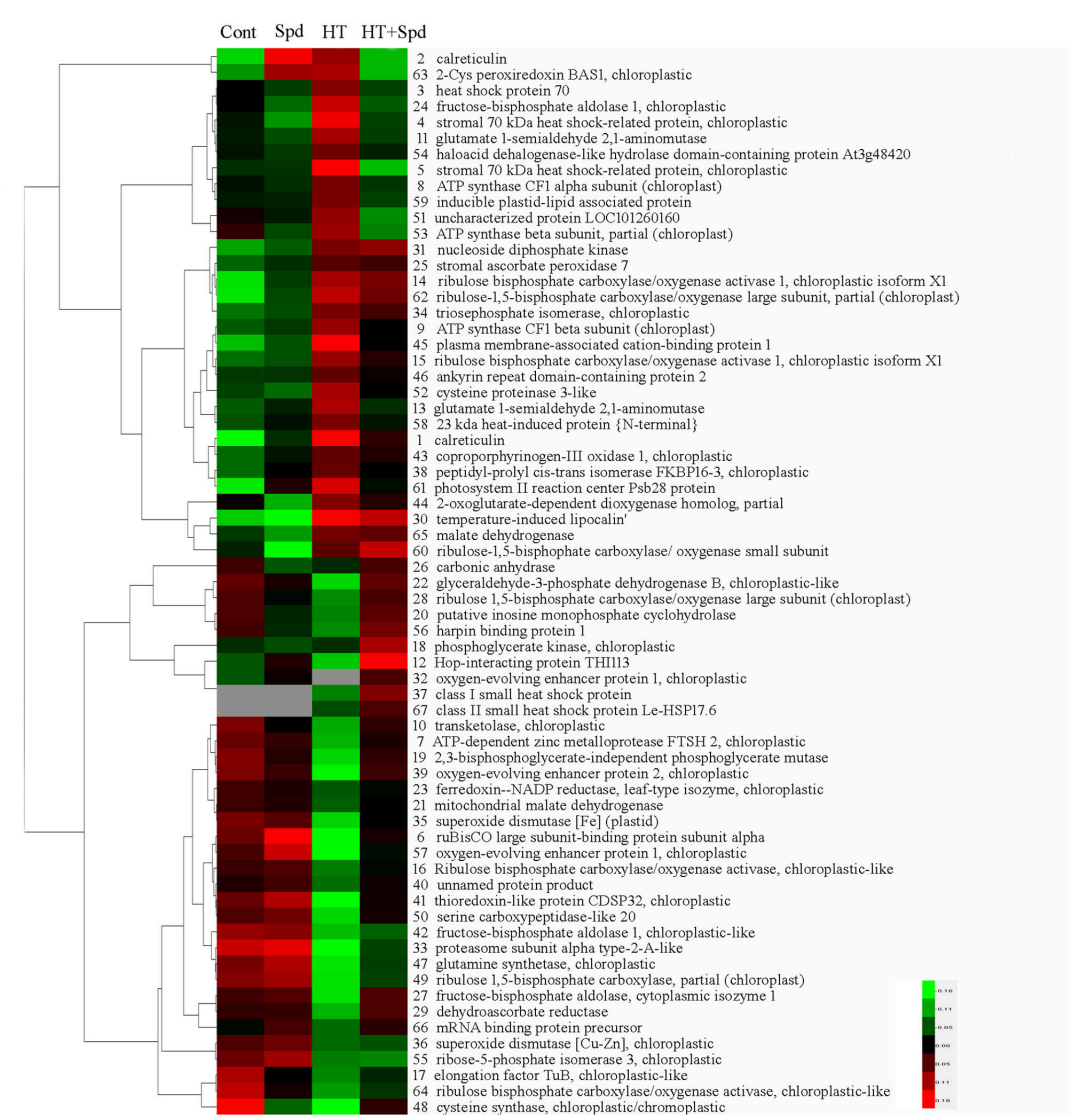
**Hierarchical clustering of differentially accumulated, tomato leaves protein spots in response to Spd and/or high temperature stress**. The four columns represent four treatments. Rows represent individual protein spots, and the protein names were labeled to the right of the corresponding heat maps. Protein spots not detected in any of the treatments are indicated in gray. Red and green show the higher and lower expression levels, respectively.

To obtain a comprehensive overview of the differentially expressed proteins, hierarchical cluster analysis was conducted to categorize the proteins that showed differential expression profiles affected by Spd under the normal and high-temperature stress conditions (Figure 5).

### Antioxidant enzymes and related genes expression analysis

**Figure 6:**
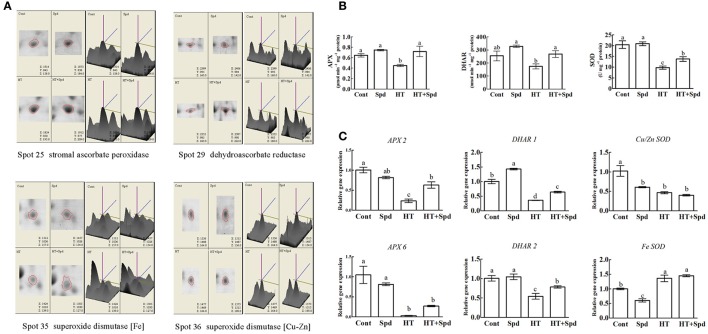
**Analysis of antioxidant responses to Spd and/or high temperature stress**. **(A)** Magnified 3D comparison of differentially expressed protein spots. **(B)** The activities of ascorbate peroxidase (APX), dehydroascorbate reductase (DHAR), superoxide dismutase (SOD) in the leaves of tomato exposed to the high temperature stress. **(C)** qRT-PCR analysis of antioxidant enzymes related genes expression. Each histogram represents a mean ±SE of three independent experiments (*n* = 3). Different letters indicate significant differences between treatments (*P* < 0.05) according to Duncan's multiple range tests.

The proteomic results revealed that the abundances of some antioxidant enzymes (spots 25, 29, 35, 36) were changed (Figure 6A), so we further analyzed the associated antioxidant enzyme activities (APX, DHAR, SOD) and related gene expressions (*APX 2, APX 6, DHAR 1, DHAR 2, Fe SOD, Cu/Zn SOD*). The activities of the enzymes showed significant decreases under high-temperature stress. However, exogenous Spd remarkably increased their activity compared with the high-temperature stress alone (Figure 6B). A similar trend was observed for the expression levels of most of the antioxidant enzyme related genes (Figure 6C).

### Interaction network analysis

**Figure 7:**
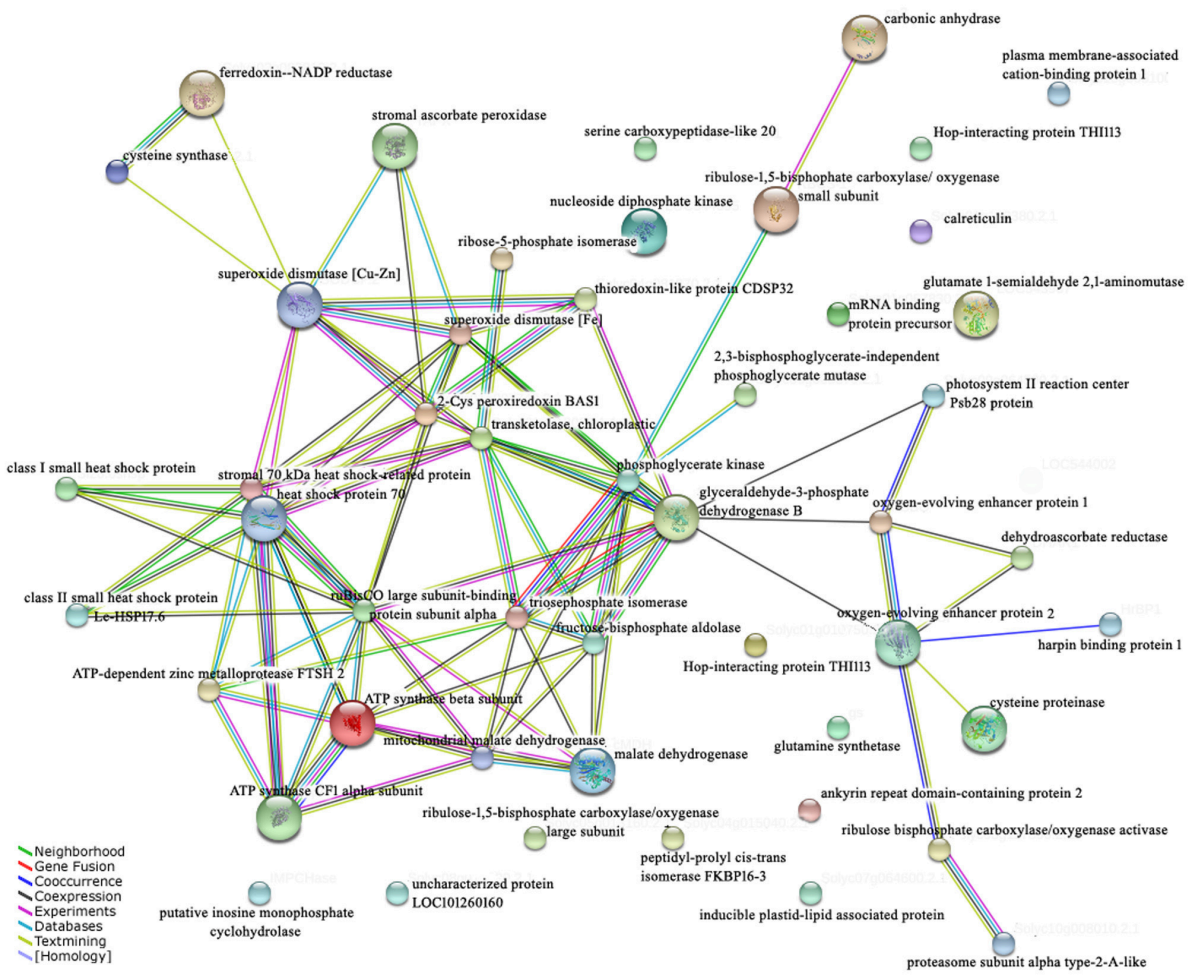
**Interaction network of the identified proteins**. Mapping of the network was performed using the STRING system (http://string.embl.de) based on confirmed and predicted interactions. Lines of different colors indicate different evidence types for the association of the proteins.

The proteins act together in the context of networks in cells, rather than performing their functions in an isolated manner (Bian et al., 2015). The STRING database provides a critical assessment and integration of protein–protein interactions, including direct (physical) as well as indirect (functional) associations. A network was used to show the interactions of the identified proteins and revealed the potential information at the protein level (Figure 7). Most energy metabolism related proteins (86.7%) and cell rescue and defense (68.8%) were involved in the protein–protein interaction network. Among the interaction proteins, the energy metabolism related proteins represented the highest proportion (35.1%). More importantly, GAPDH (spot 22) and phosphoglycerate kinase (spot 18) were the important junctions of interacting proteins in the network, suggesting that energy was of the utmost importance for the response to high temperature stress with exogenous Spd treatment.

## Discussion

Polyamines are known to effectively alleviate the plant growth inhibition by abiotic stress. In this study, exogenous Spd was shown to promote the growth and improve the photosynthetic capacity of the tomato under high-temperature stress (Figure 1), which is consistent with a previous finding in rice (Mostofa et al., 2014). 2-DE analysis was conducted, and 67 differentially regulated proteins were identified in response to high temperature and/or exogenous Spd (Figure 2, Table 1). The regulation of the metabolic processes by Spd and high temperature is discussed below.

### Photosynthesis-related proteins

Photosynthesis is highly sensitive to high-temperature stress and is often inhibited before other cell functions are impaired (Mathur et al., 2014). Importantly, Rubisco and Rubisco activase (RCA) are the primary limiting factors of net photosynthesis under stress (Ahsan et al., 2007; Hu et al., 2015). In this study, we found that proteins related to Rubisco (spots 6, 28, 49) and RCA (spots 16, 64) markedly decreased in response to high-temperature stress, similar to other proteomic studies (Han et al., 2009; Lin K. H. et al., 2015). High temperature can reduce the activation state of Rubisco (Law and Crafts-Brandner, 1999), which is often attributed to the thermolability and loss of activity of RCA under high-temperature stress (Salvucci and Crafts-Brandner, 2004; Sharkey, 2005). However, exogenous Spd had positive effects on Rubisco and RCA in tomato leaves, suggesting that the Calvin cycle and photosynthetic carbon assimilation were maintained at high levels, contributing to the biomass accumulation under high-temperature stress.

Ferredoxin-NADP reductase (spot 23) is the last enzyme in the transfer of electrons during photosynthesis from PS I to NADPH, producing NADPH for CO_2_ assimilation (Fukuyama, 2004; Tian et al., 2015). Oxygen-evolving enhancer proteins (spots 32, 39, 57) are also involved in the light reaction of PS II, and are the most heat-susceptible part of the PS II apparatus (Vani et al., 2001). The abundances of ferredoxin-NADP reductase and the oxygen-evolving enhancer (OEE) decreased in response to high-temperature stress, but the expression significantly increased with the application of Spd compared with the stress alone, suggesting that Spd played an active role in the photosynthetic chain, resulted in a higher stability of PS II and an enhancement of oxygen evolving complex capacity, and then subsequently led to an enhancement of the photosynthetic capacity (Shi et al., 2013; Su et al., 2013).

Three spots were identified as proteins implicated in chlorophyll biosynthesis. Glutamate-1-semialdehyde 2,1-aminomutase (spots 11, 13) is an important enzyme to catalyze the formation of 5-aminolevulinic acid (ALA), a vital precursor of chlorophyll (Zhu et al., 2013). Coproporphyrinogen III oxidase 1 (spot 43) catalyzes the oxidative decarboxylation of coproporphyrinogen III to protoporphyrinogen IX in the chlorophyll biosynthesis pathways (Tian et al., 2015). Interestingly, the expression of chlorophyll biosynthesis proteins was increased under high-temperature stress, whereas the chlorophyll content was decreased (Figure 1C). One plausible explanation of this observation is that chlorophyll biosynthesis in plants is very complicated and co-regulated by many factors, but the temperature-related inhibition of the enzyme activity could be an important reason for the inhibition of the chlorophyll biosynthesis.

### Cell rescue and defense

Plants have evolved a complex sensory mechanism to monitor and adapt to prevailing environmental conditions (Ahsan et al., 2007). Heat shock proteins (HSPs) are typically induced when cells are exposed to high-temperature stress, and are closely related to the acquired thermo-tolerance (Charng et al., 2006). In our study, three forms of HSP70 (spots 3, 4, 5) were identified and significantly up-regulated under high-temperature stress, which is a key part of the high-temperature response (Liao et al., 2014). In addition, two small heat shock proteins (sHSPs, spots 37, 67) were found to be newly induced by high-temperature stress, and were both found to be absent under normal conditions. The sHSPs were further up-regulated by exogenous Spd, suggesting that Spd played a crucial role in maintaining proper folding, facilitating the refolding and preventing the aggregation of the denatured proteins under high-temperature stress (Shi et al., 2013). In this experiment, the stimulation of the heat shock protein with the application of Spd may be relevant to the influence of polyamines on the DNA-binding capacity of heat shock transcriptional factor HSF (Desiderio et al., 1999).

Reactive oxygen species (ROS) metabolism is a universal response to environmental stresses. The stress-induced accumulation of ROS seriously damages the cellular membrane and internal function components, and plants have developed an antioxidant system to regulate the ROS level (Li et al., 2015). In the present study, five proteins were found to have antioxidant-related functions. Among them, Spd increased the abundances of stromal ascorbate peroxidase (APX, spot 25) and dehydroascorbate reductase (DHAR, spot 29) under high-temperature stress (Figure 6A). Further analysis revealed that the activities of APX and DHAR were increased significantly with the application of Spd under high-temperature stress (Figure 6B). The enhanced activities could be largely explained by the up-regulated mRNA levels of *APX2, APX6, DHAR1*, and *DHAR2* (Figure 6C). Interestingly, the expression of superoxide dismutases [Fe] (Fe SOD, spot 35) in the plastid was not in accordance with the superoxide dismutase [Cu-Zn] (Cu/Zn SOD, spot 36) in the chloroplast. Moreover, the protein expression, activities of enzymes and related mRNA levels also showed different change patterns in response to high-temperature and/or Spd treatment. The variance might be due to the post-transcriptional regulation and post-translational modification of SOD through complex mechanisms, which needs further study. Taken together, the exogenous Spd is involved in antioxidant and detoxification defense mechanisms, mitigating oxidative damage and intensifying the resistance to high-temperature stress (Mostofa et al., 2014; Sang et al., 2016).

### Protein synthesis, folding and degradation

Generally, abiotic stress causes a transient suppression of *de novo* protein synthesis (Capriotti et al., 2014). In this study, proteomic analysis identified two spots related to protein synthesis, including elongation factor TuB (spot 17) and mRNA binding protein precursor (spot 66), which were markedly decreased under the high-temperature stress. However, the expression was enhanced after the application of Spd. According to previous data (Li et al., 2012), it can be hypothesized that stimulating the synthesis of specific proteins by exogenous Spd may play important roles in regulating the proteins synthesis and translational machinery, which are important components of the stress response in plants.

Two proteins (spots 38, 46) that induce proper protein folding and/or prevent the aggregation of stress-damaged proteins were preferentially upregulated under high-temperature stress. In agreement with this observation, the upregulation of peptidyl-propyl *cis–trans* isomerase FKBP 16-3 (spot 38) had been reported in *Arabidopsis* and rice in response to high-temperature stress (Palmblad et al., 2008; Gammula et al., 2011). The two proteins showed a decreasing pattern under the stress with Spd, indicating that exogenous Spd might regulate protein folding and assembly, participating in the high-temperature stress tolerance.

Proteolysis is a complex process involving many enzymes and pathways in various cellular compartments. Proteases play a central role in metabolism under abiotic stress as they are involved in protein inactivation and the degradation of damaged proteins (Capriotti et al., 2014). In our study, the cysteine proteinase 3-like (spot 52) was up-regulated under high-temperature stress, in agreement with previous studies (Koizumi et al., 1993; Callls, 1995). Interestingly, the expression of ATP-dependent zinc metalloprotease (spot 7) and proteasome subunit alpha type-2-A-like (spot 33) were decreased under high-temperature stress but increased significantly with the application of exogenous Spd. Stimulating the proteolysis of specific proteins by Spd accelerated the degradation of misfolded/damaged proteins, and made tissues more stable by covalently attaching with proteins (Li et al., 2013). Furthermore, the PAs regulated the protein metabolism and may reprogram the proteome in response to abiotic stress (Yuan et al., 2016), which may also account for the resistance to high-temperature stress of the tomato seedlings.

### Energy and metabolism

It is well-known that sufficient ATP is necessary in response to abiotic stress in plants (Hu et al., 2014). Three proteins associated with ATP synthase (spots 8, 9, and 53) were significantly upregulated under the high-temperature stress, suggesting a higher energy demand for the degradation and biosynthesis of proteins (Das et al., 2015). However, the ATP synthase proteins were down-regulated by the exogenous application of Spd under high-temperature stress, stabilizing the process of ATP synthesis, and energy metabolism.

ATP is mainly produced by carbohydrate metabolism, such as glycolysis, the tricarboxylic acid cycle and the pentose phosphate pathway (Hu et al., 2015). The first group included 7 proteins involved in glycolysis pathway. Among them, our results showed that fructose-bisphosphate (FBP) aldolase in the cytoplasm (spot 27), and chloroplastic (spot 42) decreased significantly under high temperature. Moreover, glyceraldehyde-3-phosphate dehydrogenase (GAPDH, spot 22), and 2,3-bisphosphoglycerate-independent phosphoglycerate mutase (spot 19) also decreased under the stress, which would inhibit the glycolysis pathway and glycolysis associated with intermediate metabolism. However, exogenous Spd up-regulated these proteins, allowing more carbohydrates to enter the glycolic pathway and maintain the normal physiological metabolism of the tomato seedlings, thereby supporting the high-temperature resistance (Shan et al., 2016). The second group included malate dehydrogenase (MDH, spots 21, 65), involved in the tricarboxylic acid cycle. In this study, MDH showed different accumulation patterns in response to high temperature, whereas exogenous Spd sprayed on the leaves maintained the MDH expression at a high level. The third group was protein participating in the pentose phosphate pathway. Under high-temperature stress, the abundance of transketolase (TK, spot 10) and ribose-5-phosphate isomerase (spot 55) decreased. Spd application further improved the abundance of TK, whereas it affected ribose-5-phosphate isomerase unremarkably. Adjusting the EMP-TCA-PPP pathway to produce more energy may be an important mechanism for Spd to alleviate stress induced damage (Li et al., 2015).

### Signal transduction

Signal transduction pathways play an important role in abiotic stress at the cellular level, leading to changes in metabolic pathways and cellular processes. After the perception of the stress, a signal would be transferred from the cell surface to the nucleus, and then the responsive proteins would be translated (Guo et al., 2013). Within this functional category, we identified calreticulin (spots 1, 2) and harpin binding protein 1 (spot 56). Calreticulin, a major endoplasmic reticulum Ca^2+^-binding chaperone, is involved in a variety of cellular signaling pathways. Calreticulin also plays a crucial role in regulating Ca^2+^ intracellular homeostasis (Nakamura et al., 2001). In our study, calreticulin was significantly up-regulated under the high-temperature stress, but was down-regulated by Spd. These observations suggested that Spd has a relationship with the stress-induced Ca^2+^ signal transduction, probably allowing the release of free Ca^2+^ to relieve stress. Interestingly, harpin binding protein 1 was significantly down-regulated in the tomato leaves under the high-temperature stress, which was concordant with the finding in spring soybean under cold stress (Tian et al., 2015). However, the protein level recovered the controlled level after Spd treatment, and the regulatory mechanism remains unclear.

## Conclusion

**Figure 8:**
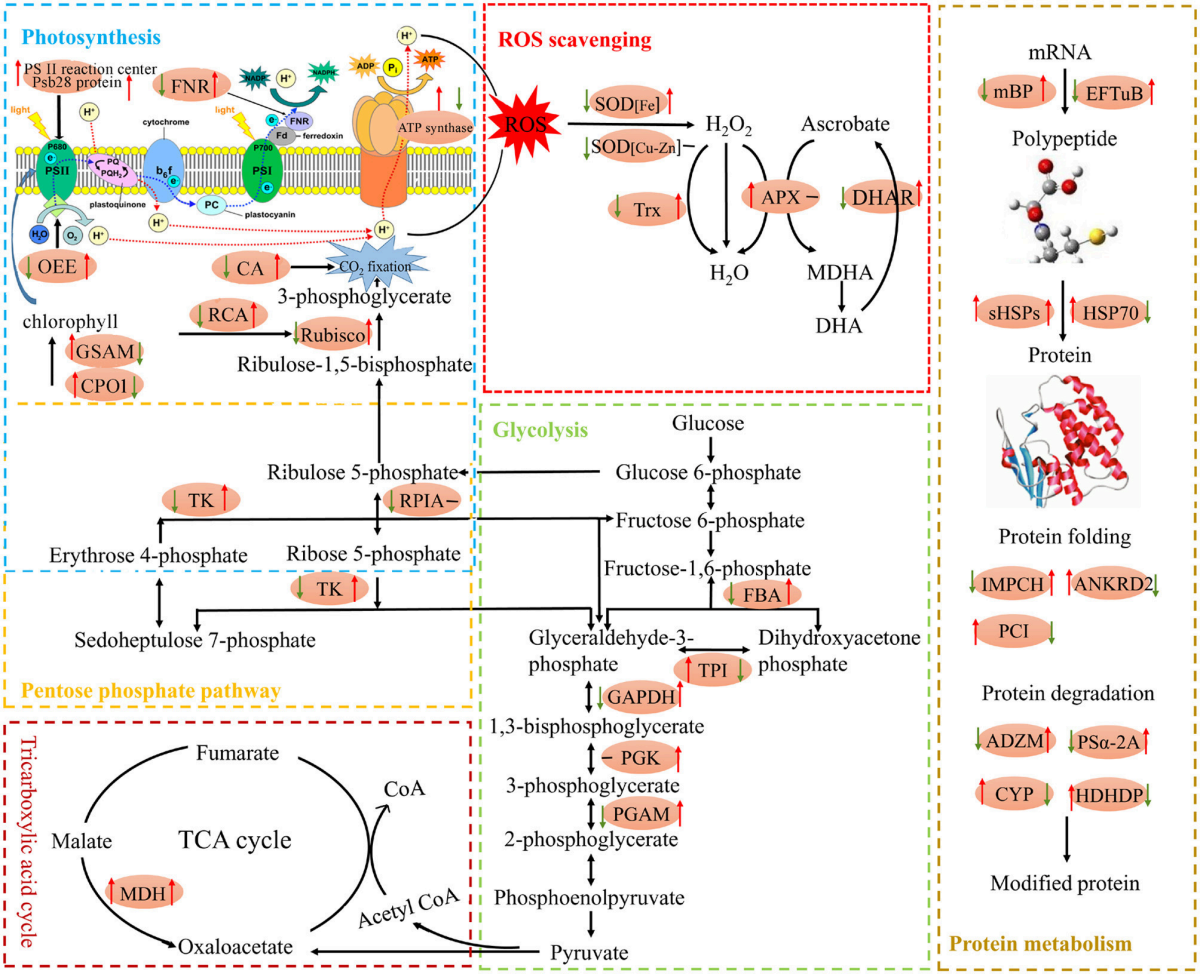
**Schematic presentation of main metabolic pathways regulated by Spd in tomato leaves exposed to high temperature stress**. Changes in protein abundance marked in red ellipse were integrated. Arrows on the life side of the ellipses indicate changes induced by high temperature stress as compared with the control, and arrows on the right side indicate changes induced by Spd under high temperature stress conditions. Red or green arrows represent up-regulation or down-regulation, respectively, and the black short lines indicate no change. ADZM, ATP-dependent zinc metalloprotease FTSH 2; ANKRD2, ankyrin repeat domain-containing protein 2; APX, stromal ascorbate peroxidase; CA, carbonic anhydrase; CPO1, coproporphyrinogen-III oxidase 1; CYP, cysteine proteinase 3-like; DHAR, dehydroascorbate reductase; EFTuB, elongation factor TuB; FBA, fructose-bisphosphate aldolase; FNR, ferredoxin–NADP reductase; GAPDH, glyceraldehyde-3-phosphate dehydrogenase B; GSAM, glutamate 1-semialdehyde 2,1-aminomutase; HDHDP, haloacid dehalogenase-like hydrolase domain-containing protein At3g48420; HSP70, heat shock 70 kDa protein; mBP, mRNA binding protein precursor; MDH, malate dehydrogenase; OEE, oxygen-evolving enhancer protein; PCI, peptidyl-prolyl cis-trans isomerase FKBP16-3; PGAM, 2,3-bisphosphoglycerate-independent phosphoglycerate mutase; PGK, phosphoglycerate kinase; PSα-2A, proteasome subunit alpha type-2-A-like; RPIA, ribose-5-phosphate isomerase 3; sHSPs, class I small heat shock protein; IMPCH, putative inosine monophosphate cyclohydrolase; SOD, superoxide dismutase; TK, transketolase; TPI, triosephosphate isomerase; Trx, thioredoxin-like protein CDSP32.

In conclusion, our results demonstrated that exogenous Spd improving tomato seedlings growth and high temperature tolerance, could be associated with the following processes: (1) stimulating protein related to photosynthesis and energy metabolism, enhancing photosynthetic capacity, providing higher energy for various metabolic processes to cope with high-temperature stress; (2) activation of cell rescue and defense response to alleviate stress induced injuries, activating the antioxidant system; (3) stimulating protein synthesis and degrading misfolded/damaged proteins induced by high temperature stress. Schematics (Figure 8) was formed to illustrate the detailed mechanism to reveal cell metabolism regulated by high temperature and/or Spd. This study provides comprehensive insights through comparative proteomics, and would be able to better enrich our understanding of the mechanism of Spd improves the tolerance of under the high-temperature stress.

## Author contributions

SG designed the research and proposed the research proceeding. QS wrote the main manuscript text. XS and YA prepared all figures and modified this manuscript until submitted. SS and JS improved the manuscript. All authors reviewed and approved the manuscript.

## Funding

This work was supported by the National Natural Science Foundation of China (No. 31471869, No. 31401919 and No. 31272209), the Jiangsu Province Scientific and Technological Achievements into Special Fund (BA2014147), the China Earmarked Fund for Modern Agro-industry Technology Research System (CARS-25-C-03), and the Priority Academic Program Development of Jiangsu Higher Education Institutions (PAPD).

### Conflict of interest statement

The authors declare that the research was conducted in the absence of any commercial or financial relationships that could be construed as a potential conflict of interest.
