# Supplementary material for: Proteomic Analysis Reveals the Positive Effect of Exogenous Spermidine in Tomato Seedlings' Response to High-Temperature Stress
Source: Front Plant Sci. 2017 Feb 6;8:120. doi: 10.3389/fpls.2017.00120 (PMC5292424; doi:10.3389/fpls.2017.00120)
Supplement: Supplementary file 1 [file Table1.DOC]

*Supplementary Material*

**Proteomic Analysis Reveals the Positive Effect of Exogenous Spermidine in Tomato Seedlings’ Response to High-temperature Stress**

**Qinqin Sang ^1^, Xi Shan ^1^, Yahong An ^1^, Sheng Shu ^1^, Jin Sun ^1,2^, Shirong Guo ^1,2^,***

**^*^ Correspondence:**

Shirong Guo

srguo@njau.edu.cn

**Supplementary Table 1**

Primer sequences for real-time qRT-PCR of antioxidant enzymes related genes.

| Gene | Acc. No. | Primer sequence (5'→3') |
| --- | --- | --- |
| *APX2* | XM_010323395 | Forward 5'- CTCTTACAGTTGCCATCA-3´  Reverse 5'- GAGCCTTAGCATAGTCAG-3´ |
| *APX6* | NM_001247702 | Forward 5'- CTCCTCTTCTTCTTCTCTTAA-3´  Reverse 5'- AATGTACTGAATCTTCCACTA-3´ |
| *DHAR1* | NM_001247893 | Forward 5'- CATCGTTGGTCTGTGTAT-3´  Reverse 5'- TCATTCTTCTATCTACTTCTCTT-3´ |
| *DHAR2* | NM_001247295 | Forward 5'- TGTCGCAAGGTATAGTAAG-3´  Reverse 5'- AGGCTATCATTCAGTCTAAC-3´ |
| *FeSOD* | NM_001246860 | Forward 5'- GCAGTCAGTATTAGGCTTA-3´  Reverse 5'- CCATCAACTACATAAACAACA-3´ |
| *Cu/ZnSOD* | NM_001247102 | Forward 5'- ATTACCGACAAGCAGATT-3´  Reverse 5'- AATACCACAAGCAATCCT-3´ |
