# Supplementary figures and images for: Proteomic Analysis Reveals the Positive Effect of Exogenous Spermidine in Tomato Seedlings' Response to High-Temperature Stress
Source: Front Plant Sci. 2017 Feb 6;8:120. doi: 10.3389/fpls.2017.00120 (PMC5292424; doi:10.3389/fpls.2017.00120)

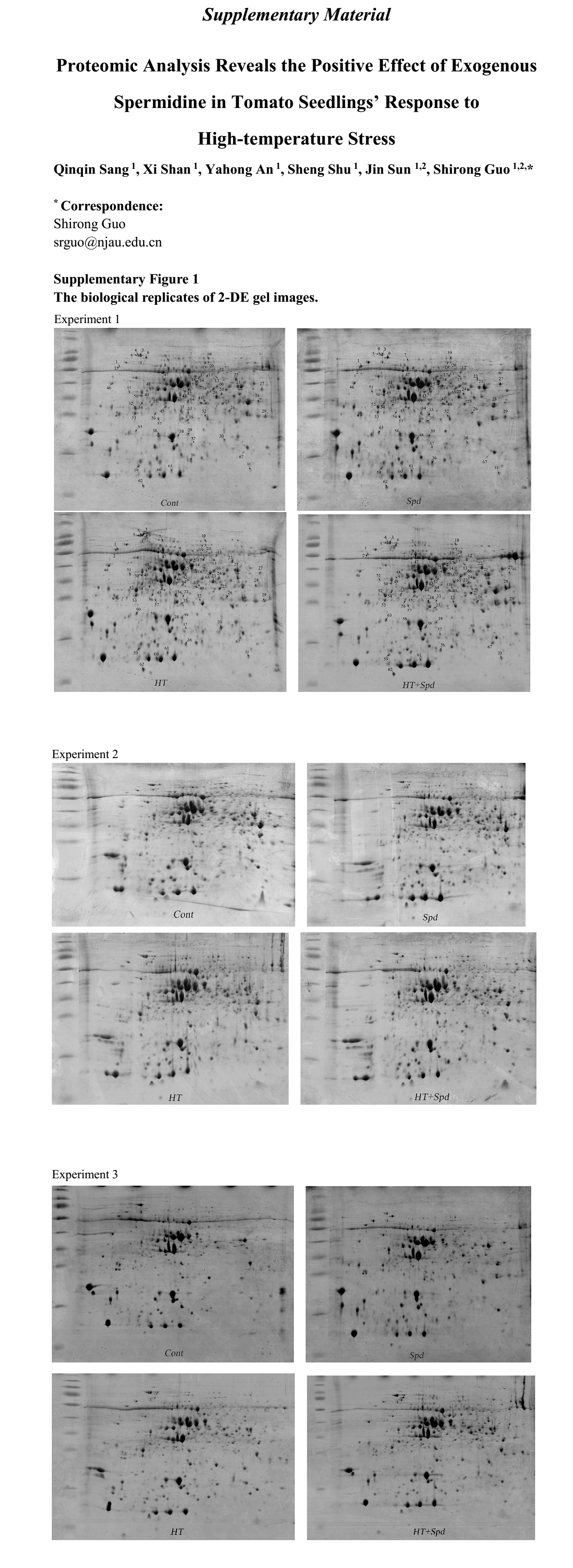

Supplement: Supplementary file 2 [file Image1.TIF]
